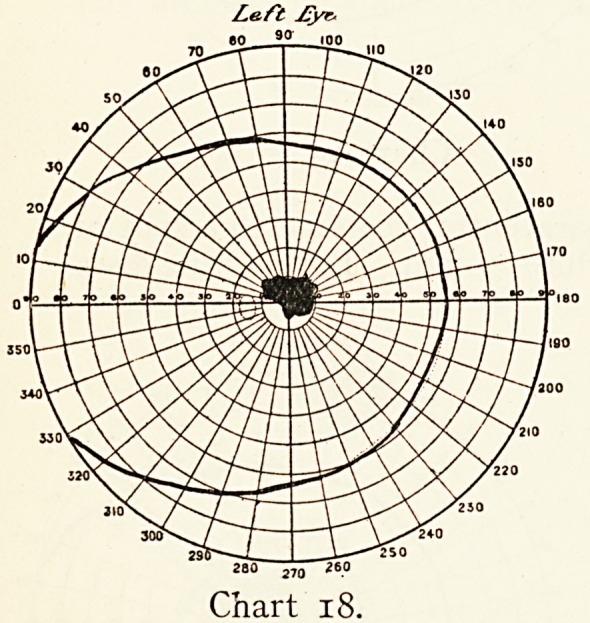# Defects in the Visual Field

**Published:** 1911-03

**Authors:** F. Richardson Cross

**Affiliations:** Consulting Ophthalmic Surgeon, Bristol Royal Infirmary; Surgeon to the Bristol Eye Hospital


### DEFECTS IN THE VISUAL FIELD.

BY

F. Richardson Cross, M.B. Lond., F.R.C.S.

*Consulting Ophthalmic Surgeon, Bristol Royal Infirmary; Surgeon to the Bristol Eye Hospital.*

---

### THE OCCIPITAL LOBE.

The late Dr. C., of Clifton, came to me on the 12th of July, 1888, complaining that he had suddenly found some serious defect of his vision. Two nights before this visit he had gone to bed well. In the morning he awoke with discomfort in the head and difficulty in seeing.

I found that with help from glasses his far sight and his reading sight were both good. There was no defect in the retina or optic nerves, nor any apparent disease in the eyes. He had an absolutely complete right-sided hemianopsia, cutting his visual field into two halves by a vertical line, which only turned slightly aside to allow of perfect vision in the axis of fixation. There was good sight on the left side of him ; on the right side he was blind. No other defect was noticed except immobility of the pupils to light coming from the right side.

Here was a case of a complete paralysis of the visuo-sensory area in the calcarine region of the mesial surface of the left occipital lobe or of the optic radiations passing to it.

Dr. C. rested only a few days and then resumed his large practice, going on with it until his death, which occurred suddenly about three years later, probably from a cerebral

Chart 1.

hemorrhage or possibly from cardiac failure. No change had taken place in the condition of the visual fields. His sight became much more useful as with time he became accustomed to the disability.

Colonel M., aged 71, came to me on October 28th, 1910, complaining that he has had some definite confusion of sight ; for nine months previous to that he had seen very well with the aid of glasses. One night he had gone to bed feeling poorly, and the next morning he found his sight confused. He was ill for a few days with headache and indefinite malaise. The doctor considered it a case of influenza. He was soon able to see to read with little difficulty and to get about in comfort, though his sight was not as it had been prior to his illness.

I found far sight with correction was 6/9 in each eye, and he could read the smallest print. There were a few striæ in the crystalline lenses, but the optic nerves and fundus showed no defect. At first there appeared to be nothing wrong, but a very careful examination of the visual fields detected a small symmetrical left-sided hemianopsia, not noticed by him, as it lay in the upper left field, where vision is but little used. When the defect was pointed out to him he at once realised it as the sole cause of his discomfort.

Chart 2.

These two cases illustrate the extremes of a complete and a partial one-sided blindness, both uncomplicated, coming on suddenly and remaining unaltered. Such hemianopsias may occur without being necessarily an early symptom of further brain trouble. I have seen a large number of patients who with more or less defect in their visual brain centres, as shown by a permanent unaltering hemianopsia, have been able to do useful work apparently without discomfort, and who have remained without any other evidence of disease for many years.

Many cases like those quoted are quite uncomplicated. I believe they are due to an embolism of a branch of the calcarine artery. The onset is usually sudden : the patient has some slight brain disturbance for some days and then recovers, except that the obstructed brain area undergoes atrophy, and its visual function is permanently lost. There is little or no evidence throughout of any defect in the optic disc.

In others there may be present a very slight paralysis somewhere on the same side of the body as that of the blind area, but the damage is quite localised and remains so.

Dr. S., aged 73, consulted me on February 1st, 1899. On December 31st, 1898, he had gone to bed well. In the morning he woke up with slight left hemiplegia. When I saw him he seemed well except for a slight numbness of the left hand. Vision was *R.* 6/6, *L.* 6/9, with slight left hemianopsia ; optic nerves slightly pallid, but practically normal. The interesting point in this case was that ten years previously, while taking a bath, he was seized with headache and vomiting. For three weeks he lay insensible, and for some time after he was practically blind in both eyes. He had no kind of paralysis or other

Chart 3.

symptom. Rather suddenly his sight cleared. He felt poorly for six months, but after that he was quite well, and continued to practise until the later attack in 1899. It is uncertain whether the hemianopsia was associated with the attack in 1889 or in 1899.

Of course, hemianopsia may be a part of a spreading lesion, an early symptom of thrombosis, which will lead to progressive softening, or of a tumour which is causing pressure upon the visual cortex or radiations, or upon the optic tract, but here we should have optic neuritis and other symptoms. I am speaking of cases where the sight area only is impaired.

I have taken charts of all kinds of quadrantic defects, both sides quite symmetrical, and always reaching the periphery of the fields, either confined to one quadrant or more or less implicating both. A most interesting one is shown.

Mrs. H., aged 71, a patient of Dr. Statham, seen April 10th, 1909. Two years and a half before she had a slight cerebral attack after influenza with a little weakness of the left side; a second attack quite recently had caused more weakness of the left leg and arm. When I saw her vision was with correction R. 6/9 and good reading, L. 6/36. The left leg could not be lifted freely, the left arm felt heavy, but there was neither definite defective sensation nor paralysis; there was a little want of expression of the left side of the face.

The weakness of the body was on the left side; there is a double quadrantic hemianopsia of the left side. We may assume that each of the two slight cerebral attacks mentioned was associated—one with the defect in the upper quadrant of

Chart 4.

the two visual fields, the other with the defect in the lower quadrants.

In the one we may assume a lesion in some part of the lingual gyrus or below the calcarine fissure; in the other a lesion above the calcarine fissure in the cuneate gyrus. For probably the superior portion of the retina is associated with the cuneate gyrus and the median aspect of the occipital lobe above the calcarine, while the lingual gyrus below the calcarine is associated with the lower part of the retina, a lesion here causing loss of vision in the upper quadrant of the visual field.

### THE CHIASMA.

The optic chiasma lying on the groove of the sphenoid would seem to be easily implicated in inflammation or distension of the sphenoidal cavities or of the cavernous sinus, while its close proximity to the pituitary body renders it very liable to be influenced in diseases of that organ.

The anterior lobe of the pituitary is a closed gland, the secretion from which appears to have an inhibitive action on growth. The disease called acromegaly is probably due to the defective function of this gland, any swelling of which is nearly always associated with defect in the function of the more central fibres of the chiasma; these go towards the nasal side of the retina, and when damaged cause impairment in the temporal fields of vision (bitemporal hemianopsia).

The defect in the fields varies much according to the degree of pressure on the chiasma, and with the way in which the optic

Chart 5.

nerves or tracts may be implicated. Chart 5 shows a typical hemianopsia with acromegaly in which the symptoms were slight, while in a case I showed at the Neurological Society, [1] in which all the typical symptoms of acromegaly were very marked, the impairment of the visual field was throughout rather a right-sided hemianopsia than a bitemporal one. With improvement of the patient's health under thyroid extract, there was a gradual removal of pressure from the nerve fibres and almost complete recovery of the field of vision.

I have seen several well-marked bitemporal hemianopsias in which there was no suggestion of acromegaly.

1 *Brain*, 1902, xxv. 341a. Many charts in this paper.

In one a lady was under observation for six years : throughout was a widely-distributed defect of the visual fields of a distinctly bitemporal type. Sometimes the patient was pretty well, but occasionally suffered from severe symptoms of intracranial pressure, headache, severe vomiting, and impairment of central vision, once or twice to almost complete blindness. During thsese paroxysms she always had double optic neuritis ; then the symptoms would pass off, and for a time she was pretty well, with pallid, non-swollen nerves, and with good central vision of one eye. The temporal areas of the visual

Chart 6.

fields were permanently affected, though the degrees of implication somewhat varied. At one time her condition seemed hopeless ; she was nearly blind with great pain in the head and sickness ; trephining was urged, but declined. Soon after this the "materies morbi," probably a tubercular cyst (not connected with the pituitary body), which was pressing on the chiasma, etc., dried up, and she rapidly recovered her health, and for the past five years she has been quite well, excepting that she still has definite unaltering bitemporal hemianopsia with atrophic pallor of both optic nerves. The central vision is only seriously impaired in one eye. The cure has been attributed to Christian Science.

Chart 6, January, 1902, selected from among many taken between 1900, the commencement of the illness, and 1906, when permanent improvement took place.

Miss P. came on September 12th, 1904, complaining that the right eye had for some months felt uncomfortable with defective sight, varying in degree, at times preventing her from reading for days together. The central vision of the right eye with correction was 6/18 J12, that of the left was normal; but the fields showed well-marked bitemporal hemianopsia in the upper quadrants passing downwards. (See Chart 7.)

The optic nerves were pallid, especially the right. The charts suggested marked pressure on the chiasma, probably on its lower part, and on the right optic nerve, possibly from abnormity at the body of the sphenoid or the third ventricle. There were absolutely no symptoms of acromegaly and no optic neuritis.

Chart 7.

There was an indefinite history of illness with catarrh of the eyes or influenza as a possible cause. I saw the patient again on October 20th, when the right vision had further slightly deteriorated.

On December 6th, 1909, in answer to inquiries, Dr. Delpratt Harris kindly wrote: "Yes, Miss P. is still living, and fairly well but for her bitemporal hemianopsia, congestive headaches, at times lasting about ten days, which completely prostrate her and reduce her sight. During the headaches she gets severe vertigo, which prevent her from moving without discomfort, even in bed. She has absolutely no localising symptoms, paralysis or defective sensations. Vision does not seem altered, but I am never allowed to use the ophthalmoscope or perimeter, which she says make her worse. She is very irritable."

This interesting case shows how chronic such a condition may remain without progressive nerve deterioration and without any evidence of implication of the pituitary gland.

THE INTRACRANIAL PORTION OF THE OPTIC NERVES.

The optic nerves in their intracranial portions, and in the foramen, and apex of the orbit, are in such close proximity to the sphenoidal sinus or the posterior ethmoidal cells, on one or both sides, that they might be expected to participate in diseased conditions of these structures. In cases of caries, necrosis, or suppuration they could scarcely escape, nor in thrombosis of the neighbouring veins, and a very large number of cases of defective eyesight have been published as the result of diseased conditions of the sinuses accessory to the nose : in such, examination of the papilla and of central and peripheral vision may assist in localising the disease.

But grave sinus mischief may be present without any material damage to the sight, while on the other hand vision may be from this cause impaired, when serious disease in the sinus is not very evident. We should expect some narrowing of the peripheral field, possibly bitemporal, rather than a loss of central vision. Even when the sight is defective, it is not often easy to detect any definite change in the optic disc, because the actual disease is at such a long distance from the end of the nerve in the eyeball—for it seems obvious that the farther up from the papilla a given lesion exists, the less marked are the changes likely to be as seen by the ophthalmoscope. As an instance of the existence of serious mischief to the optic nerve without any evidence at the papilla, we know that sometimes a fall on the head is at once followed by blindness more or less complete of one eye (assumedly from fracture towards the optic foramen), and that in these cases a descending optic atrophy will show itself in a few weeks, though early examination detects no change whatever in the appearance of the optic disc.

I think in many cases of sinus trouble there is no definite change at the optic disc, no evidence of interference with the circulation in the nerve, or at its edge. There may be a shallowing of the physiological cup, a little pallor of the disc, or slight haze around it, but occasionally there may even be a definite papillœdema.

Mrs. E. C. H., aged 41, had an operation in July, 1902, on the left nasal fossa followed for some days by severe neuralgia over whole ophthalmic nerve area. In October temporary blurring of the sight of the left eye. On February 11th, 1903, sudden complete loss of the sight of the left eye. After a month's interval she could see a candle flame, and in August saw fairly. She came to me March 29th, 1904. *Vision : R.* normal, *L.* 6/18, J 10 ; field full nearly, slight central colour defect. No difference in the appearance of the two optic discs or eye fundus. The blindness had been probably due to retro-ocular neuritis.

Florence K., aged 26, came to the Eye Hospital on October 1st, 1908, complaining that the sight of the right eye had been rapidly failing for fourteen days. There was no perception of light in the eye ; the sight of the left was perfect. On October 6th the sight of the left was failing, and on the 10th she could only count fingers with that eye. She was admitted into the Hospital.

There was no optic neuritis, the vessels were somewhat attenuated on the discs, especially on the right, but the appearances were practically normal. There was no pain, and no head symptoms. She was leeched on the temple, given calomel, mercurial inunction, etc.

It seemed as though some inflammation of the right optic nerve far back was spreading to the other side. The sight was so defective it was impossible to test the field of vision. The condition was suggestive of sinus trouble, but no other symptoms of it could be discovered. In five or six days the sight improved to *R.* hand movements, *L.* 2/60. On October 24th fingers could be counted with each eye, but only in the nasal halves of the fields of vision ; in the temporal fields she was blind.

For several days the sight fluctuated, and once there was no perception of light in the left eye, and very severe headache came on. On November 1st the right papilla was very pale, the left œdematous, with slight exudate on the veins, and some retinal hemorrhages. There was also slight sickness. The left eye was blind, the right saw fingers in the nasal field.

November 8th. Getting salivated and is rather better. Nov. 12th. Vision in each eye 1/60. *Right,* optic disc pale ; *left,* optic disc slight neuroretinal haze but clearing, exudates and hemorrhages absorbing. The conditions continued to improve, and she was discharged at her own wish on December 13th.

She had been very ill, and was practically blind for several days. No definite diagnosis could be made. Although mercurial treatment seemed to have been useful, there was no reason for suspecting syphilis. When discharged vision was *R.* 5/60, *L.* 1/60.

On January 21st, 1909, *R.* 6/12, *L.* 6/36. Fields full central and peripheral.

March 31st, 1910. Patient says she is perfectly well, and has been so for nearly a year. *R.* 6/9, J1., *L.* 6/18 J4; fields full; optic nerves white (atrophied); pupil movements sluggish.

Both eyes had failed rapidly, one preceding the other by only three weeks. After a serious illness of three months improvement was quite definite, and continued until really good vision was restored, though the appearance of the optic nerve is that of atrophy.

### THE ORBITAL PORTION OF THE OPTIC NERVE.

The almost complete fixation of the optic nerve within the skull, and at the apex of the orbit, is in marked contrast with the freely movable condition of the trunk of the nerve as it lies in orbital cavity.

The orbital portion is quite free and loose, not straight but shaped like an S, to allow free rotation for the back of the eyeball. The nerve thus readily escapes pressure, particularly as there is room for considerable swelling in the large bony cavity that contains it.

Exophthalmic goitre shows to what an extent the nerve can be stretched forward without any impairment of the sight, or any fault in the appearance of the optic disc.

I lay great stress in the diagnosis of the position of an intra-orbital swelling, on (1) the appearance of the papilla (2) the implication of the field of vision, as evidence of direct pressure on the optic nerve or not.

There may be gross lesion in the orbit without any defects in the visual fields or at the optic disc.

Changes at the disc are probable where the lesion is not far behind the eyeball.

The term **retro-ocular or retro-bulbar neuritis** is applied to a group of cases in which defective vision is due to impairment in the orbital portion of the optic nerve outside the eyeball. It may be due to pressure *on the outer surface of the nerve trunk* from such causes as cellulitis, periostitis, syphilis, tubercle, tumour, etc. Or the cause may lie *within the nerve,* among its actual fibres. Gout, rheumatism and influenza may be factors in its causation, and it is not unfrequently associated with disseminated insular sclerosis. It may follow renal disease, high arterial tension, and diabetes. And I have seen cases of fairly typical retro-ocular neuritis with no defect recognisable by the ophthalmoscope show at a later stage definite hemorrhages at the optic disk. Retro-ocular hemorrhages may take place instead of neuroretinal hemorrhage.

Chart 8.

I append a few charts of the visual field taken from a very large number of similar cases. Those bounded by a crossed line are the field taken with red colour.

When Dr. Arthur Wood, of Hereford, was my private assistant we carefully tabulated a large number of my perimeter charts, taken in cases of typical optic neuritis and optic atrophy. In all of them the field was narrowed at the periphery as in Chart 8, which is really, however, hysteria in boy, and only in very few was there any definite central scotoma; the latter seemed to be generally cases of locomotor ataxy. Chart 9 shows one of these.

Mr. G., aged 50. No history of syphilis. In 1873 fell from a scaffolding; insensible for twelve hours. Apparently recovered completely, and made no complaint of his sight until 1901. I saw him March, 1902. Typical A.R. pupils, knee-jerks absent, neuralgia in legs. Vision, *R.* 1/60, J20, *L.* 6/60 J19.

Both fields are narrowed at the periphery: in the left there is a wide area of blindness just reaching the fixation point; the right periphery is only slightly affected, but there is an absolute central scotoma.

In retro-ocular neuritis the blindness is nearly always central; indeed, the existence of a central scotoma with absence of disease near the yellow spot of the retina, and no defect in the optic disc, is almost pathognomonic of the disease in question.

Chart 9.

In acute cases the condition is usually **monocular**—a rapid, severe loss of vision, unaccompanied by any other evidence of disease; slight pain sometimes along the nerve trunk.

The majority soon and almost completely recover sight, but some descending changes in the optic nerve may appear later, especially if progress is slow, and a few cases do not recover.

*Acute Retro-Ocular Neuritis (? Hemorrhage).*

Mrs. H., aged 45, saw me on May 10th, 1904. A week previously she had suddenly lost the sight of the left eye during a paroxysm of great mental excitement. The left eye scarcely saw hand movements; the area of appreciation in the field of vision was confined to a small patch on the temporal side of the field (Chart 10).

Fundus normal, except for a slight indefinite haze of the optic nerve and of the retina just around it.

The right eye with correction saw perfectly well.

Chart 10.

May 27th. She saw much better; with the right eye closed she could walk about the room and observe objects, but barely read J20: a most interesting change had occurred in the visual field (Chart 11): the periphery had quite cleared up, but there was still an absolute scotoma for white 20 deg.

Around the fixation point she had good perception of colours in the portion of the field clear at the first visit. Slight neuritis of optic nerve. July 26th, the sight of left is almost restored. Vision with correction, 6/9 J1; field for white quite perfect. Red field, full, slightly dull in centre. Optic nerve pale, arteries somewhat narrowed.

Though we usually find central scotoma as typical of an early stage of these cases, some of them commence, as in this case, with a still wider area of blindness which soon clears up peripherally.

Chart 11.

*Retro-Ocular Neuritis Depending on Anæmia.*

Miss K., aged 32, came on December 22nd, 1899, complaining of the right eyesight having failed. Vision was reduced to J19 6/0, and there was a positive scotoma for white, the red field also being very imperfect. (See Chart 12.)

There was no definite lesion to be seen with the ophthalmoscope. The patient was very anæmic; the optic nerves pallid to an equal degree. On December 26th the vision had improved to 6/60 and J10. The field was very much improved for red, but a scotoma was still present. On February 13th, 1900, the patient had completely recovered.

I considered the case to be one where the papillo-macular bundle of the optic nerve was suffering from malnutrition.

Chart 12.

*Retro-Ocular Neuritis from Hemorrhage into Axial Nerve Fibres.*

Mrs. A., aged 72, came May 16th, 1904, complaining of rapid loss of sight in the left eye. The right was nearly blind from cataract. The left had undergone a most successful extraction by Sir H. Swanzy four years previously. She was entirely dependent on this eye, with which she had seen and read perfectly. The sight had become very defective; there was a positive central scotoma, and the periphery of the field for red was narrowed (Chart 13). The media were quite clear, the eye fundus was readily seen; the retina and macula were healthy; the optic nerve was pale and hazy and senile with narrow vessels, on it was a small capillary hemorrhage, which did not account for the loss of sight. This had increased a few days later, and I now

Chart 13.

assumed a similar mischief in the axis of the nerve farther back. The patient was gouty, and frequently complained of flushings to head. After a course of baths, under Dr. Cave, she returned with the intraocular hemorrhages almost gone, and sight with glasses improved to 6/12 and J4. There remained an indefinite scotoma to a 1 mm. white spot and a 5 mm. red spot. Later she still further improved.

**Retrobulbar neuritis** may be **binocular.** The attack may be simultaneous in both eyes, or one may be first affected and the other follow at a longer or shorter interval.

The symptoms are commonly seen in tobacco amblyopia—central scotoma in the red field and more or less defective vision. Prognosis in these cases is uniformly good. The following case is unusual and instructive.

Chart 14.

The Rev. B., aged 55, consulted me May, 1898, for failing sight. The left eye had been nearly blind for a year. Vision, *L.* barely J20, large absolute central scotoma (see Chart 14); *R.* 6/9 J1 with glasses, field seems normal. He smoked a quarter-pound of shag weekly, but as the right optic nerve was normal and the left white, I thought he was suffering from left optic atrophy, gave him a hopeless prognosis for this eye, and said the right was good. In three months, however, he returned with the vision of left reduced to 6/36 and J16 and a scotoma in the field for red (see Chart 15). I now stopped his tobacco entirely, but still considered the left eye hopeless. A year later vision was, *R.* 6/8 J1, *L.* 6/24 J8, and both eyes continued to improve, though the left nerve remained atrophied. The loss of the central vision as against peripheral in the left eye should have lead me to suspect tobacco as the cause even of such severe monocular blindness, and to have given a more accurate prognosis.

The most serious aspect in binocular retrobulbar neuritis when uncomplicated is illustrated in the two following cases, of which I give charts (16 and 17).

Charles N., aged 29, came to the Bristol Eye Hospital on June 4th, 1905. There had been difficulty in reading in February. He had been a heavy smoker; this had been stopped for some months, but sight had gone on getting worse. When I saw him vision was reduced to 3/60 and J 19 in each eye, but he found his way about readily. He had no pain in the eyes or head (Chart 16). The periphery of the fields of vision were full, but he had a paracentral scotoma to a spot of red 10 mm. and to a spot of white 5 mm. Both optic discs

Chart 15.

Chart 16.

showed marked pallor on the temporal side, but were hyperæmic on the nasal side with slight swelling towards the retina. He was treated with mercurials, iodides, strychnine, etc., without any change in the condition of his sight.

November, 1910. He has now atrophy of both discs, a definite central scotoma for white, though he sees red and blue centrally in 2 mm. spots. He says the letters are not black enough to see, his vision being only 4/60 and J18 in each eye.

Alice S., aged 50, came in May, 1909, to the Bristol Eye Hospital complaining that for some weeks the sight was failing in the right eye ; for a month she has had pain behind it, and the sight is reduced to hand movements. Right eye, 1D 6/60.

In July vision in the left still perfect, right sees 6/60. The periphery of the visual field is much clearer than the centre ; no definite abnormity of the optic disk or any part of the eyeball.

On October 1st the right eye was much the same, but the left was painful and sight hazy.

On October 19th vision of left reduced to 1D 6/18, and on October 26th this eye had no perception of light. The optic

Chart 18.

Chart 17.

disc was slightly hazy, and the case diagnosed as a retro-ocular neuritis in the two eyes. She was admitted as an in-patient, and was treated with oleate of mercury and hyd. c. creta.

By November 9th she was salivated, and on the 16th sight had slightly improved to *R.* 6/60, *L.* 3/60. On November 24th vision much the same. The Charts 17 and 18 showed an absolute central scotoma. The eyes were blind to red and green. There was a fair field for blue and a good one for white, excepting centrally. She left the hospital that day.

November, 1910. Her vision now is still only *R.* 3/60, *L.* 6/36 ; the periphery of the fields is full for white, but the central scotoma remains. Both discs show well-marked atrophy.

Both these patients get about and do ordinary work without discomfort, but they cannot read, do any refined work, or sew; they will probably not improve now, having atrophy of the axial fibres of the optic nerves, but they are not likely to get worse.

---

### THE NATURE AND TREATMENT OF CHOREA.

## Figures and Tables

**Chart 1. f1:**
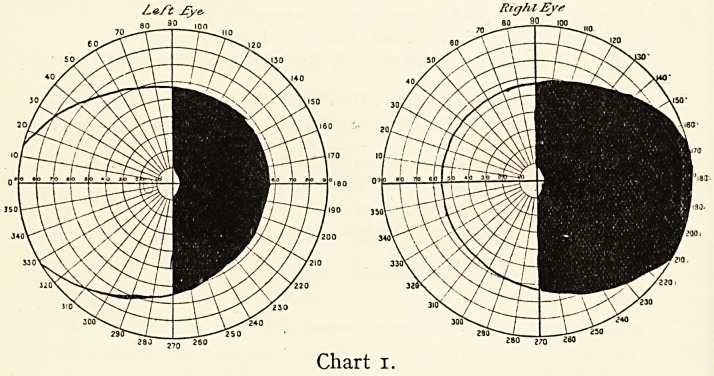


**Chart 2. f2:**
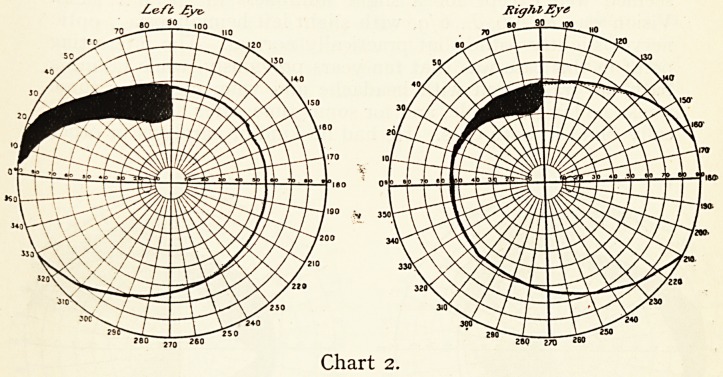


**Chart 3. f3:**
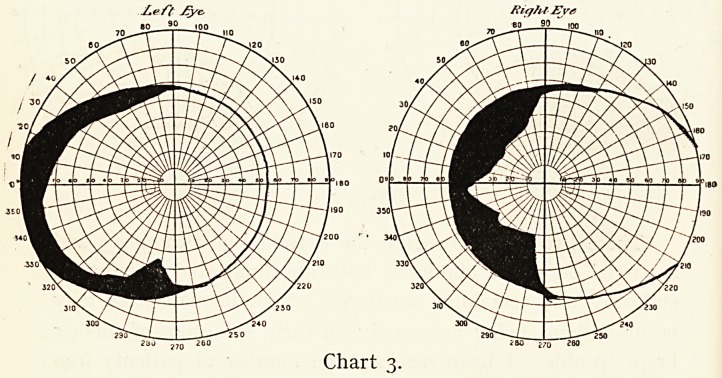


**Chart 4. f4:**
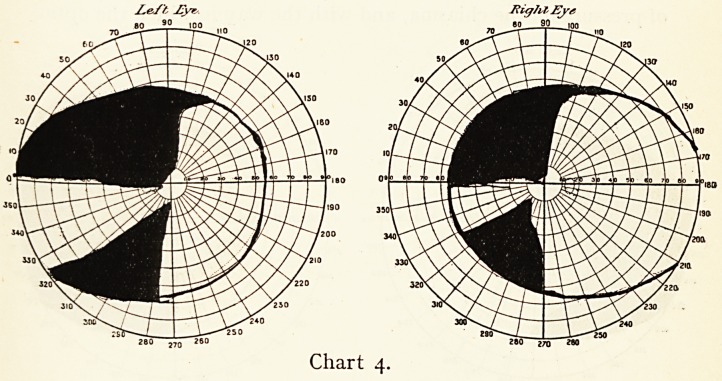


**Chart 5. f5:**
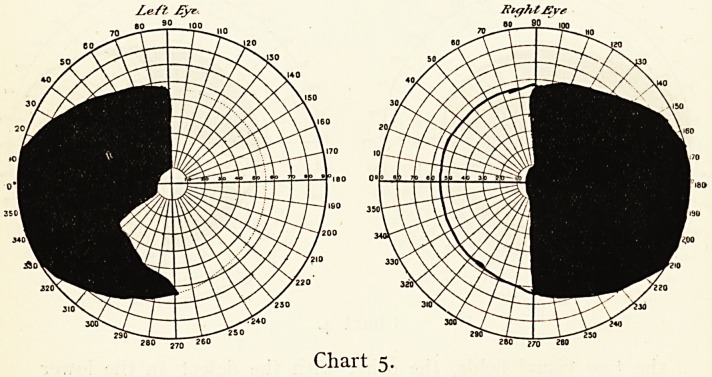


**Chart 6. f6:**
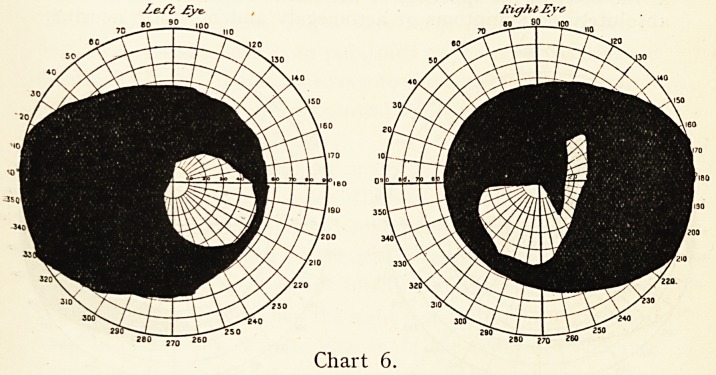


**Chart 7. f7:**
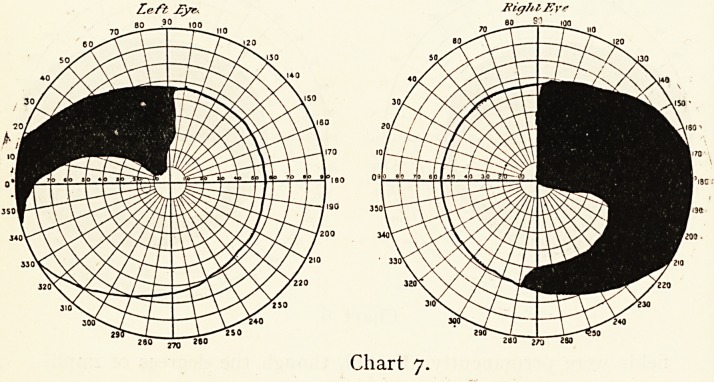


**Chart 8. f8:**
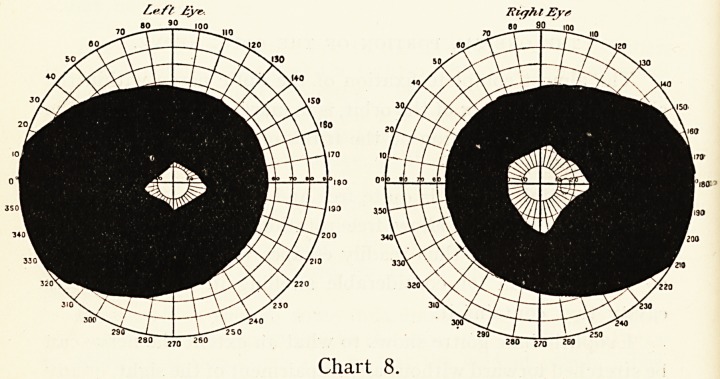


**Chart 9. f9:**
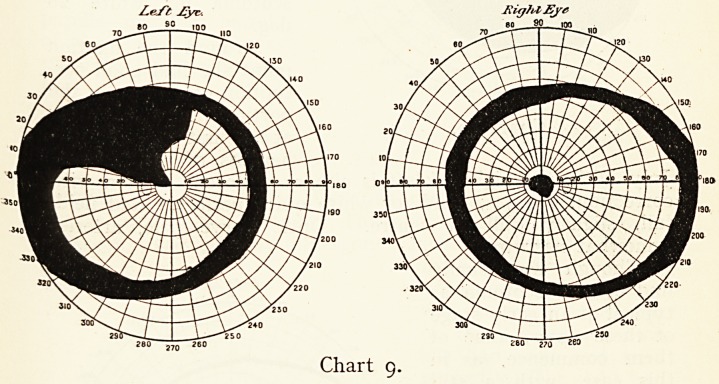


**Chart 10. f10:**
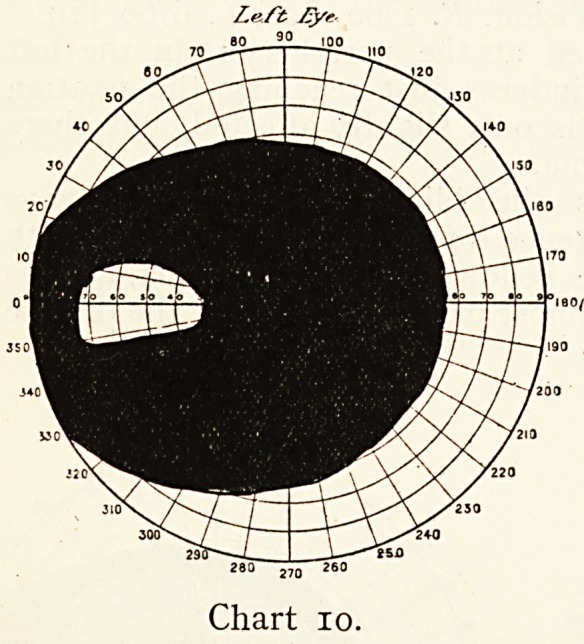


**Chart 11. f11:**
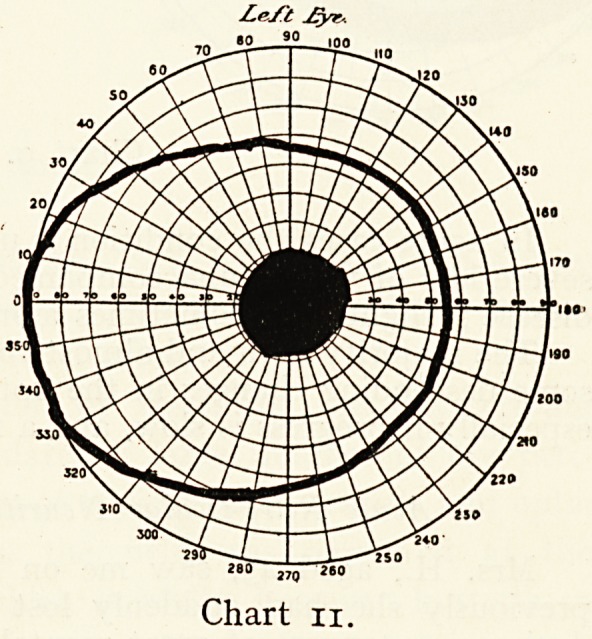


**Chart 12. f12:**
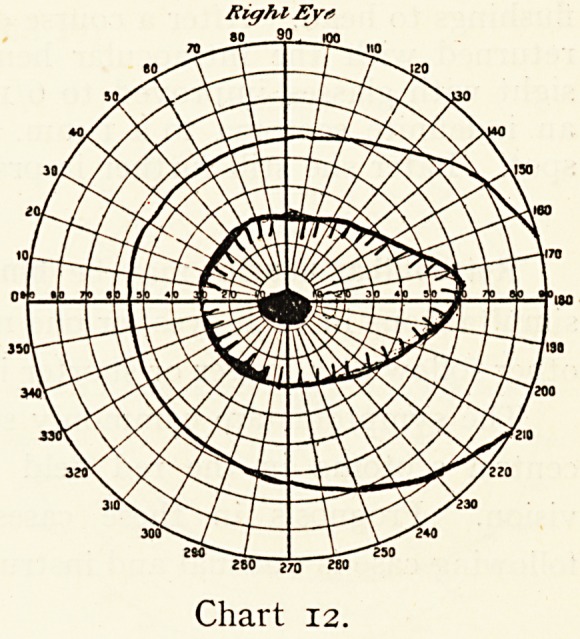


**Chart 13. f13:**
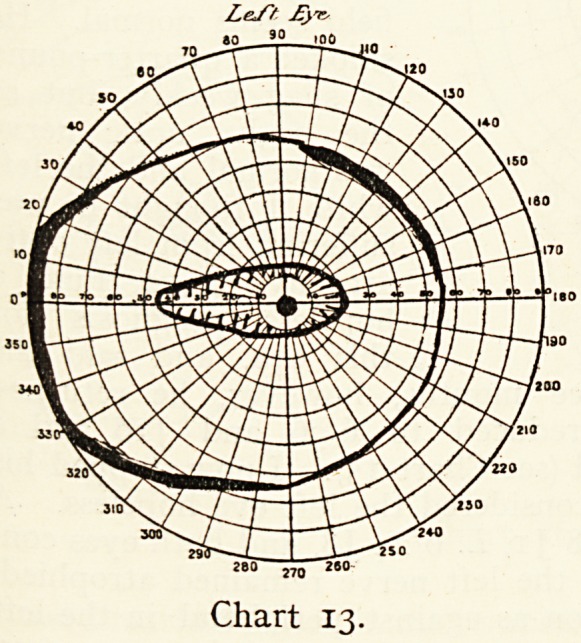


**Chart 14. f14:**
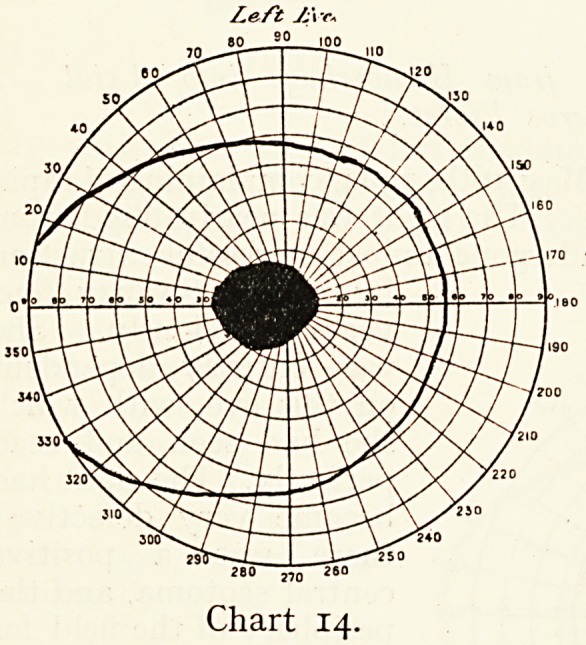


**Chart 15. f15:**
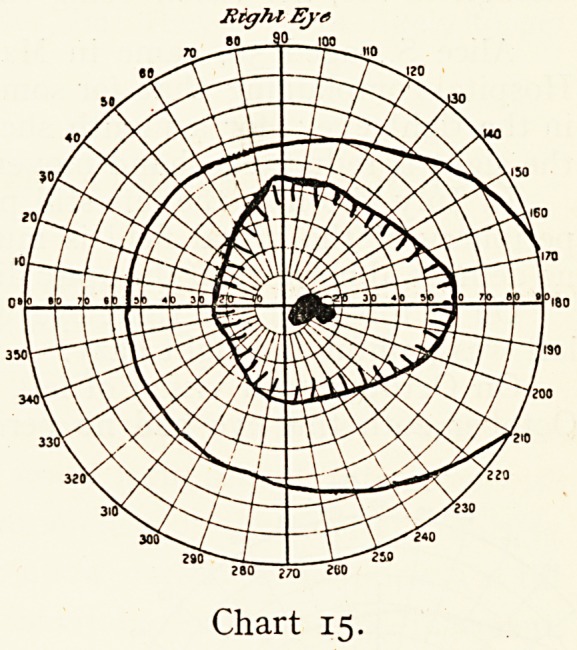


**Chart 16. f16:**
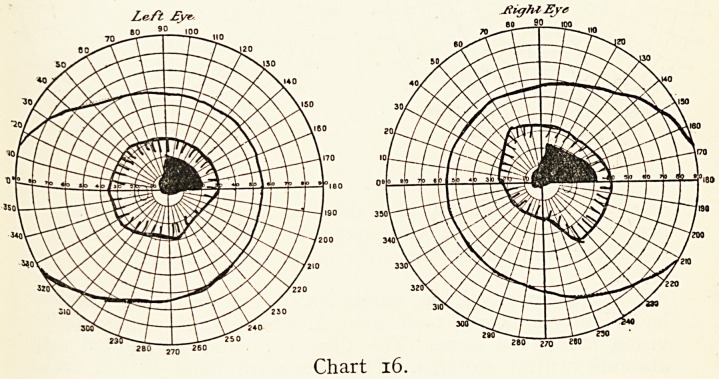


**Chart 17. f17:**
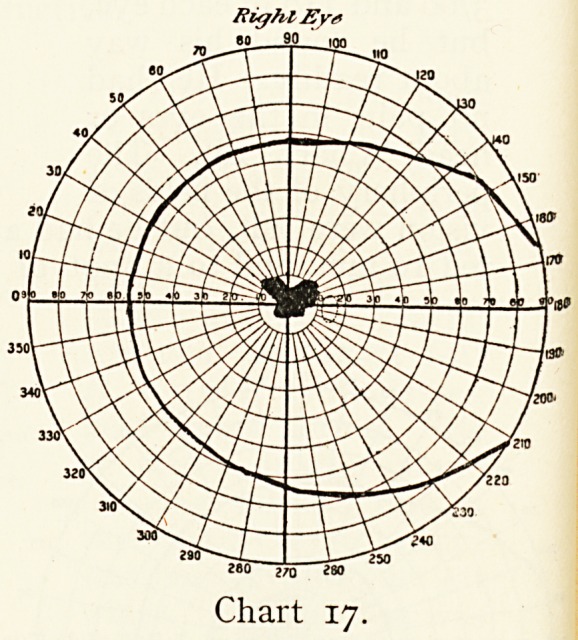


**Chart 18. f18:**